# Partial Extraction Therapy-Assisted Immediate Implant Placement in an Ellis Class VIII Fracture in the Esthetic Zone: A Clinical Case Report

**DOI:** 10.7759/cureus.94756

**Published:** 2025-10-16

**Authors:** Rohini Parui, Sri Sasank Annaluru Tejaswee, Arpita Singh, Kabir Dash, Dinesh Senapati

**Affiliations:** 1 Department of Public Health Dentistry, Kalinga Institute of Dental Sciences (KIDS) Kalinga Institute of Industrial Technology (KIIT) Deemed to be University, Bhubaneswar, IND; 2 Department of Oral and Maxillofacial Surgery, Kalinga Institute of Dental Sciences (KIDS) Kalinga Institute of Industrial Technology (KIIT) Deemed to be University, Bhubaneswar, IND

**Keywords:** aesthetic rehabilitation, bone preservation, ellis class viii fracture, implant placement, partial extraction therapy, socket shield

## Abstract

Partial extraction therapy (PET) is a surgical approach aimed at preserving the periodontium by maintaining part of the patient's tooth root during implant placement. This case report presents the detailed clinical management of a female patient diagnosed with an Ellis Class VIII fracture involving the right upper lateral incisor (tooth #12). The treatment involved the application of PET followed by implant placement, ensuring optimal esthetics and function while preventing alveolar ridge resorption. This case highlights that in the esthetic zone, instead of using the conventional implant placement method, the PET technique, when employed, resulted in a favorable emergence profile and optimal esthetic outcome within just six months. This case was documented to contribute additional evidence to the growing body of literature supporting this novel technique.

## Introduction

One of the most commonly used treatment approaches in managing Ellis Class VIII fracture is routine implantology, which involves tooth extraction followed by either immediate or delayed implant placement. The benefits of immediate implant insertion include shorter treatment durations, but this does not stop the changes in ridge dimensions that come with tooth extraction [1-3].

Disruption of the highly vascular periodontal ligament (PDL) system is a critical step in tooth extraction [4]. Once the PDL is lost, the alveolar bone is deprived of its primary source of biomechanical stimulation and biochemical signaling, both of which are essential for maintaining bone homeostasis [4]. As a result, osteoclasts are activated and initiate bone resorption, particularly targeting the bundle bone [4]. This process leads to a rapid and progressive resorption, with the most significant dimensional changes occurring within the first three to six months post-extraction [4,5]. It may lead to an esthetic and functional failure if the supporting tissues at an implant site resorb and are exacerbated by recession risk factors. Implant site recession involves the apical migration of the peri-implant mucosal margin, leading to exposure of the implant abutment or restoration surface. It is primarily influenced by factors such as a thin gingival phenotype, insufficient keratinized mucosa, buccal bone dehiscence, mal-positioned implants, and surgical or prosthetic trauma. The condition may compromise both the esthetic and functional outcomes of implant therapy [5].

In order to address the problems brought on by extraction, partial extraction therapy (PET) was developed [6]. PET includes a series of steps that employ the tooth itself to compensate for the loss of alveolar tissue [6]. In 2010, Professor Hürzeler presented PET, which is a treatment approach based on keeping a piece of the root at the coronal third of the alveolar socket, for rapid implant placement. It was suggested that this would lead to improved soft and hard tissue esthetics and increased patient satisfaction [7,8]. Here, the tooth root attachments to the bone are maintained, which maintains the PDL-bone complex and its vascularity [7]. A number of publications by several researchers have described retaining roots as an effort to lessen the unfavorable remodeling of the alveolar bone [9,10].

PET is growing more and more popular among clinical experts worldwide. This technique has demonstrated extremely promising outcomes in avoiding alveolar ridge resorption in situations involving immediate implantation post-extraction [6]. This report presents the clinical and radiological results of a case involving an esthetic implant that was treated using the PET concept.

## Case presentation

Case description

In June 2024, a 56-year-old medically fit female patient presented to the dental clinic with a chief complaint of a broken upper right lateral incisor (#12), resulting from trauma sustained during an accidental fall. The patient expressed significant concerns regarding both esthetics and masticatory function, as the affected tooth was located in the esthetic zone.

Pre-operative assessment

**Figure 1:**
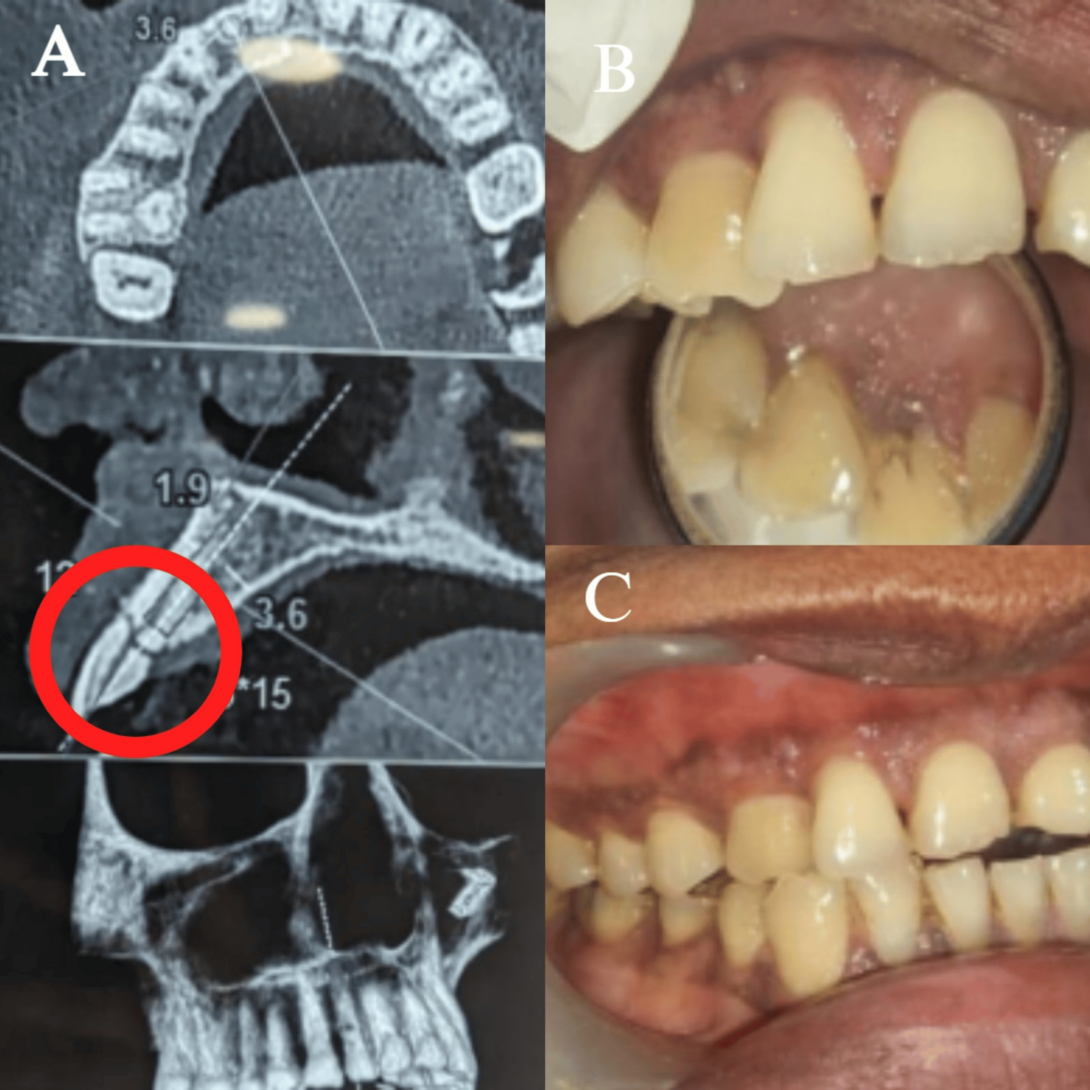
(A) CBCT scan showcasing Ellis Class VIII fracture; (B, C) intraoral images showcasing discolored tooth (#12) (A) CBCT scanned image in relation to #12, which shows a fracture line at the cervical region; (B, C) clinical image of the fractured, discolored tooth CBCT: cone-beam computed tomography

**Figure 2:**
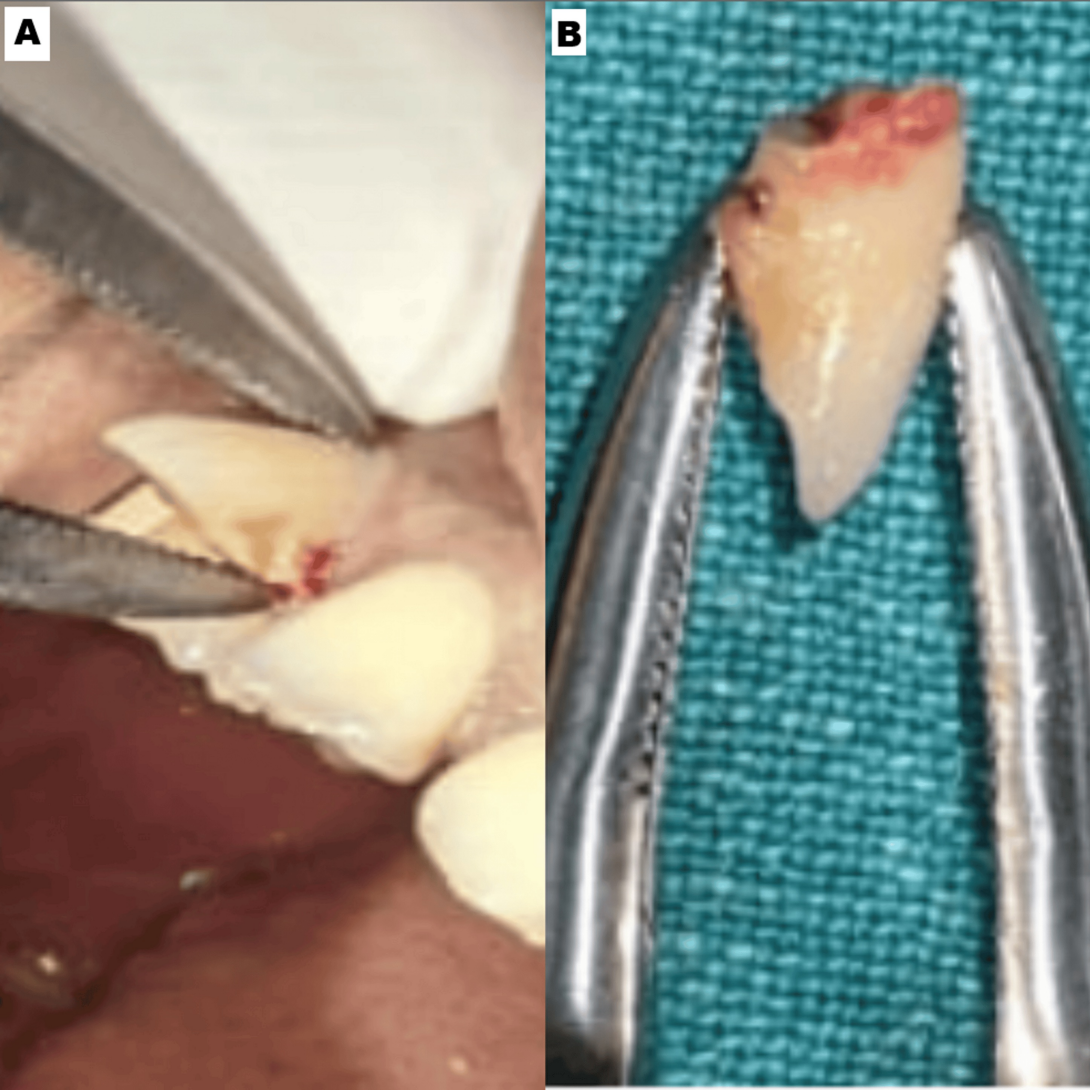
(A, B) Extraction of the fractured crown portion of tooth #12 The figures here showcase the extraction of the fractured crown in relation to #12, indicating to conserve the root

Clinical examination revealed a discolored, fractured crown in relation to the left upper lateral incisor with no tenderness to percussion, but exhibited mobility of the crown fragment (Figure 1). Radiographic evaluation, including periapical radiographs and cone-beam computed tomography (CBCT), confirmed an Ellis Class VIII fracture involving complete separation of the clinical crown and an obliquely fractured root extending sub-crestally. The fracture pattern rendered the tooth non-restorable (Figure 2).

CBCT imaging further demonstrated intact surrounding bone with adequate alveolar ridge width (6.2 mm) and height (16 mm), with no evidence of periapical pathology or bone dehiscence (Figure 1). The patient had excellent oral hygiene and no medical conditions that could adversely affect wound healing or osseointegration, making her a suitable candidate for future implant therapy. According to the esthetic risk assessment [11], the patient's strong esthetic demands exacerbated poor lip line and thick phenotype (Figure 1) [11].

Treatment plan

In the anterior region, resorption of the labial bone plate after extraction could cause soft tissue contraction and esthetic concerns; therefore, PET was utilized in this treatment plan by retaining the labial root to preserve bone and enable immediate implant placement [12,13]. CBCT scans provided us with the pre-operative radiographic assessment, and with the help of it, the implant planning for the case was done.

Details

Following a discussion with the patient about various treatment choices, an implant-supported prosthetic replacement was chosen as the preferred treatment option. An attempt was made to combine the advantages of immediate implant insertion and PET by performing partial root extraction followed by immediate implant placement. Consent was acquired after discussing all the details with the patient.

Phase 1: Partial Extraction and Immediate Implant Placement

The following armamentarium was used (Table 1).

**Table 1:** Armamentarium used in performing the treatment

Armamentarium
Surgical straight handpiece (NSK, Japan)
Straight fissured carbide bur (Hudens Bio Burstar HP Carbide Taper Fissure Burs, Korea)
Prima Dental Round Surgical Carbide Bur FG 25 mm
GDC Extraction Forceps Upper Anteriors - 1 Standard (FX1S)
GDC Periosteal Elevator P8d - 6
Waldent Curettes Lucas Bone Curette
Dio UFII Implant kit (kit composed of drill for UFⅡ Narrow Ø3.0 Ø3.3/UFⅡ Regular Ø3.8 Ø4.0 Ø4.5 Ø5.0 Ø5.5 Fixture)
UF(II)Narrow Fixture Ø3.3 × 13 mm and cover screw; UF(II)Narrow Cemented abutment
Chlorhexidine gluconate 0.2%
Local anesthetic spray: Stim Lidayn Spray
Icpa Xicaine Local Anesthetic 30 mL (Lignocaine Hydrochloride I.P. 2%, Adrenaline I.P. 1:80,000)
Bone graft
HMD Dispovan 2 mL Syringe with Needle
HMD Dispovan 5 mL Syringe with Needle
White Geistlich Bio-Oss 0.5 cc Pack (Small Particles) Bone Graft Granules

Following the application of the local anesthetic, the anterior forceps (GDC Extraction Forceps Upper Anteriors - 1 Standard (FX1S)) were used to remove the crown segment (Figure 2). After exposing the root segment, the root was sectioned mesiodistally using a straight fissured carbide bur (Hudens Bio Burstar HP Carbide Taper Fissure Burs, Korea) in a surgical straight handpiece (NSK, Japan). Radiographs were taken for evaluation. Using the periosteal elevator, the palatal root part was luxated and subsequently removed, leaving the labial segment.

**Figure 3:**
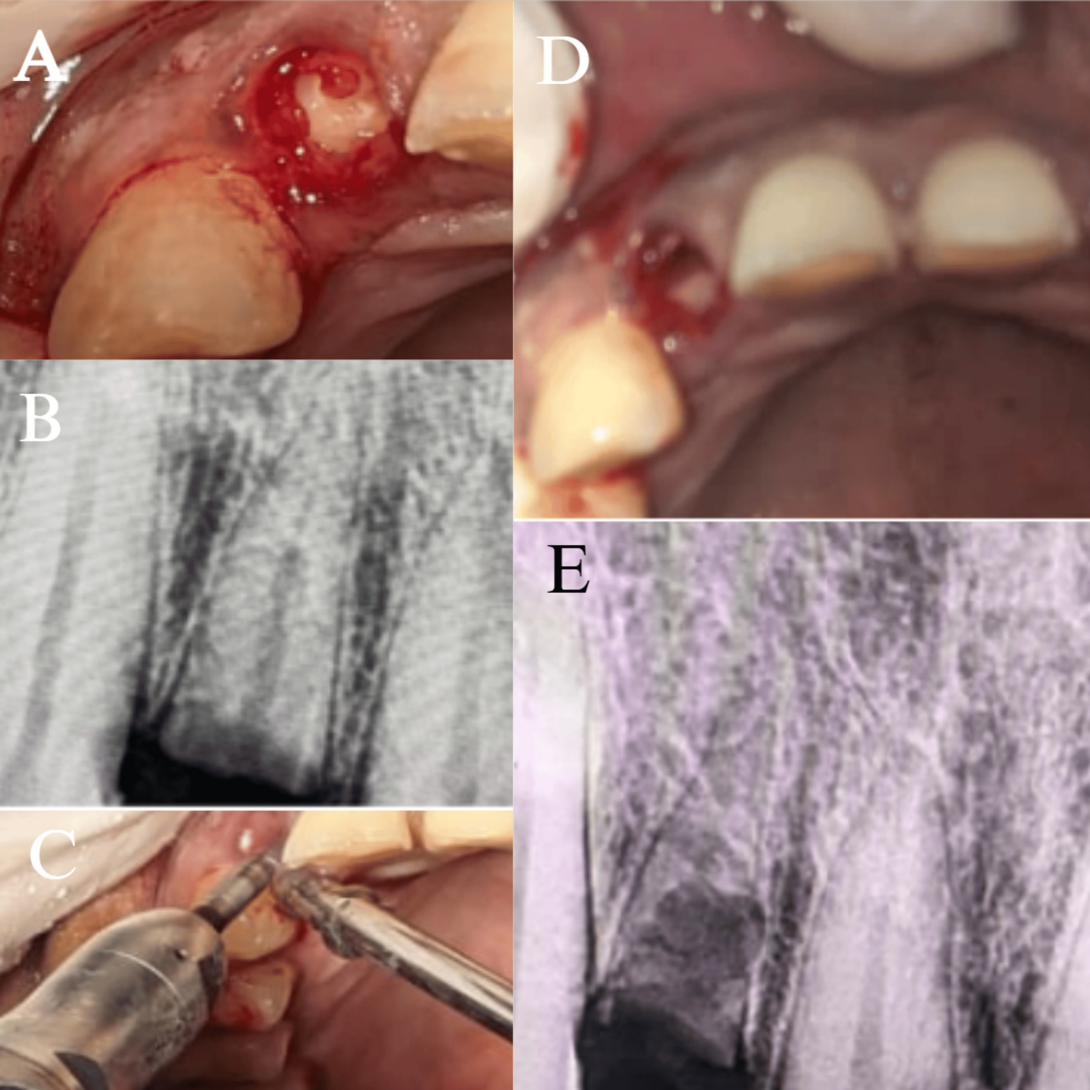
(A) Exposing the root segment of #12; (B) radiograph of the root segment; (C) sectioning of the root segment; (D) intraoral image of the labial root segment; (E) radiograph of the sectioned labial root segment (A) shows the clinical image of the root of #12, whereas (B) shows the radiographic image of the root portion of #12. (C) shows the sectioning of the root portion; (D) shows the intraoral image of the labial segment of the root after removal of the palatal portion after sectioning; (E) shows the radiographic image of the remaining labial portion of the root of #12

Round Surgical Carbide Bur FG 25 mm (Prima Dental) was used to reduce this labial fragment into a "c" shape that was just coronal to the facial alveolar bone crest in order to prevent the bony crest from being compromised during preparation. To help prevent exposure to the external shield, the fragment's facial margin was beveled, and its palatal margin was thinned and concavely curved to create prosthetic space and prevent exposure to the internal shield. The socket was debrided and disinfected with 0.2% chlorhexidine gluconate for prompt implant insertion (Figure 3).

**Figure 4:**
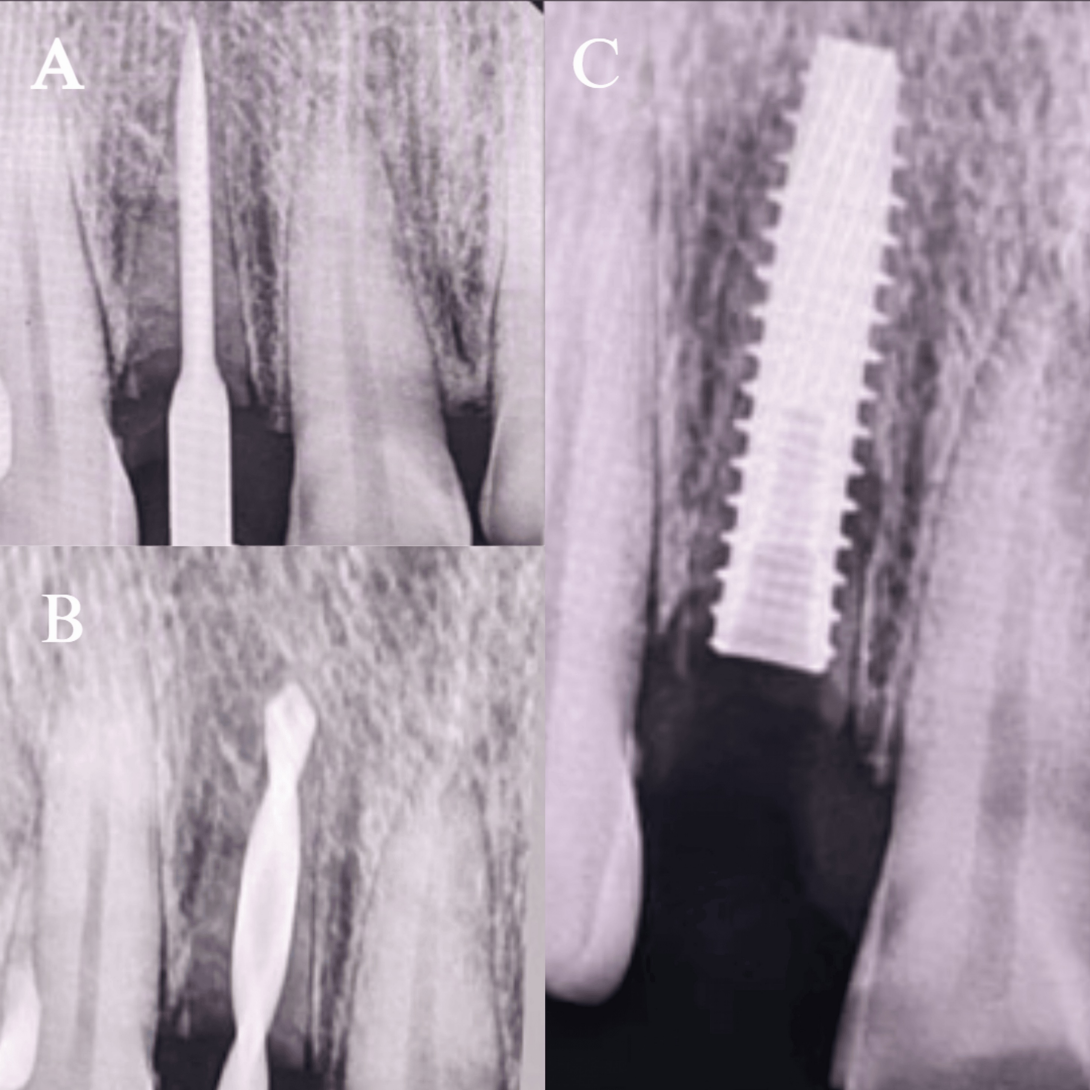
Implant placement in relation to #12 (A) is the radiograph showing the initial placement of the Starter drill; (B) is the radiograph showing the depth of placement with the last osteotomy drill. (C) shows the radiograph post-implant placement

**Figure 5:**
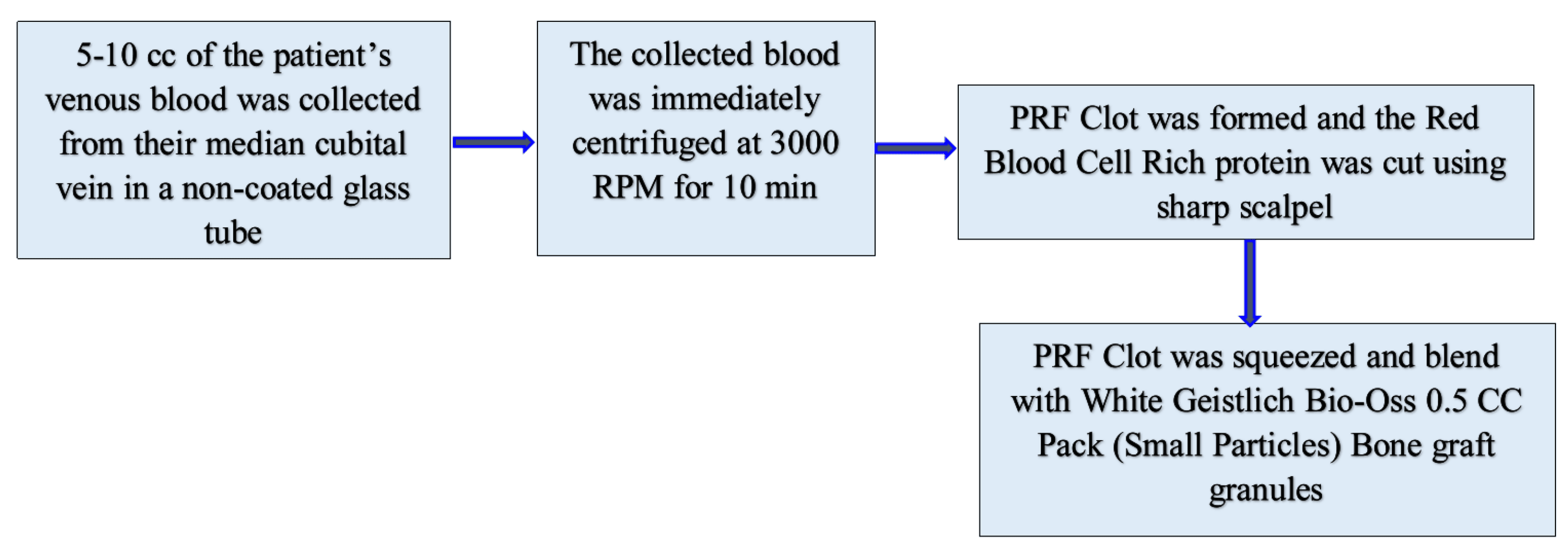
Preparation of PRF PRF: platelet-rich fibrin

To enable a prosthetically driven 3D implant placement, the osteotomy was meticulously prepared sequentially (UF(II)Narrow Fixture Ø3.3 × 13 mm, Dio Implant, South Korea). The last osteotomy drill (Ø3.3 mm) was utilized before implant to maximize the amount of "press fit" and improve the primary stability of the implant. The ISQ was 68 labiolingually and 72 mesiodistally, and the insertion torque that was attained was 35 Ncm (Figure 4). Before suturing with 3-0 Mersilk sutures (Ethicon, Inc., Somerville, NJ, US), a pack of bone graft (White Geistlich Bio-Oss 0.5 cc Pack (Small Particles) Bone Graft Granules) and platelet-rich fibrin were placed in the jumping area (Figure 5). Medications were administered, and post-operative instructions were given.

**Figure 6:**
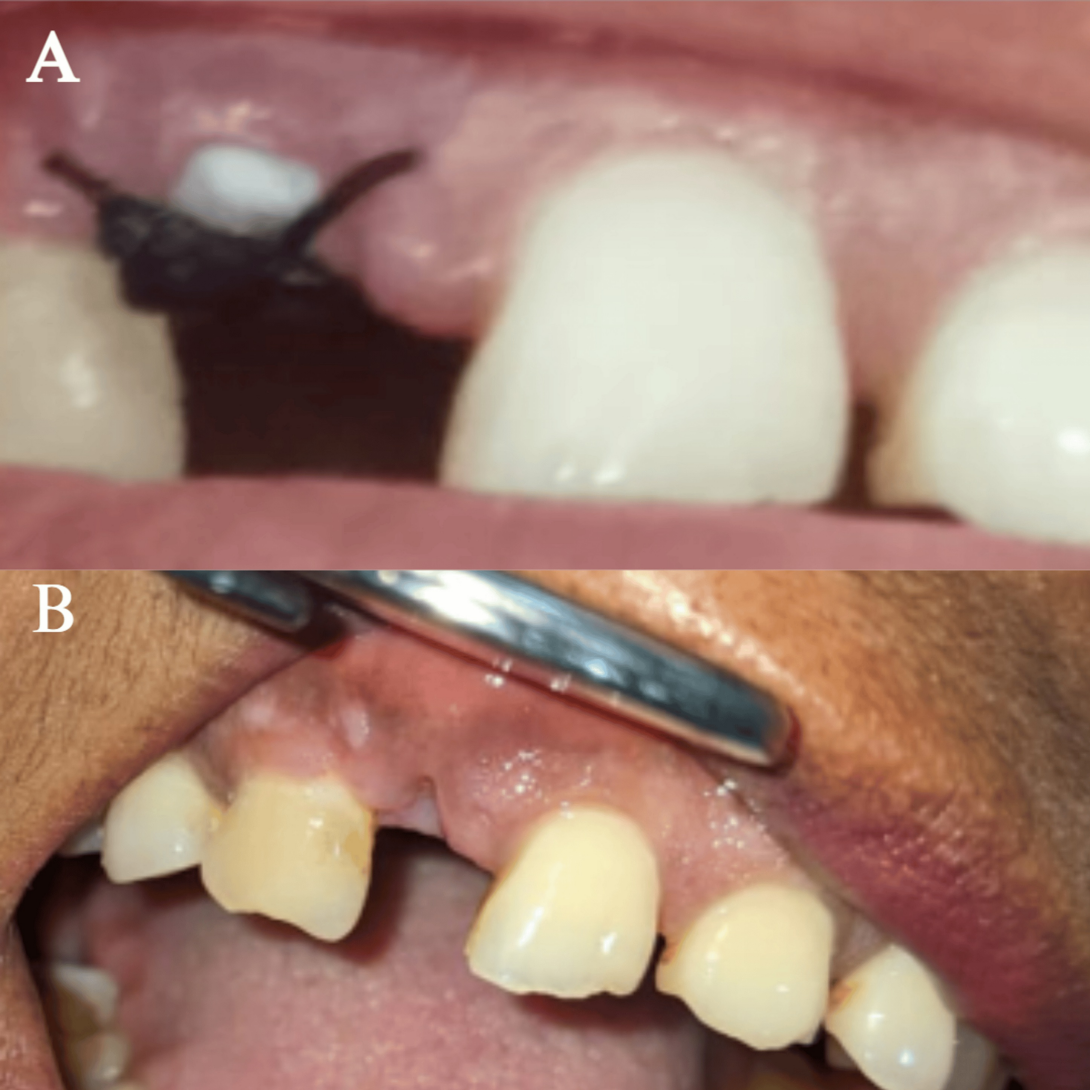
(A) Healing after 1-week follow-up; (B) healing after 2-week follow-up (A, B) show healing after one week and two weeks post-op, respectively

**Figure 7:**
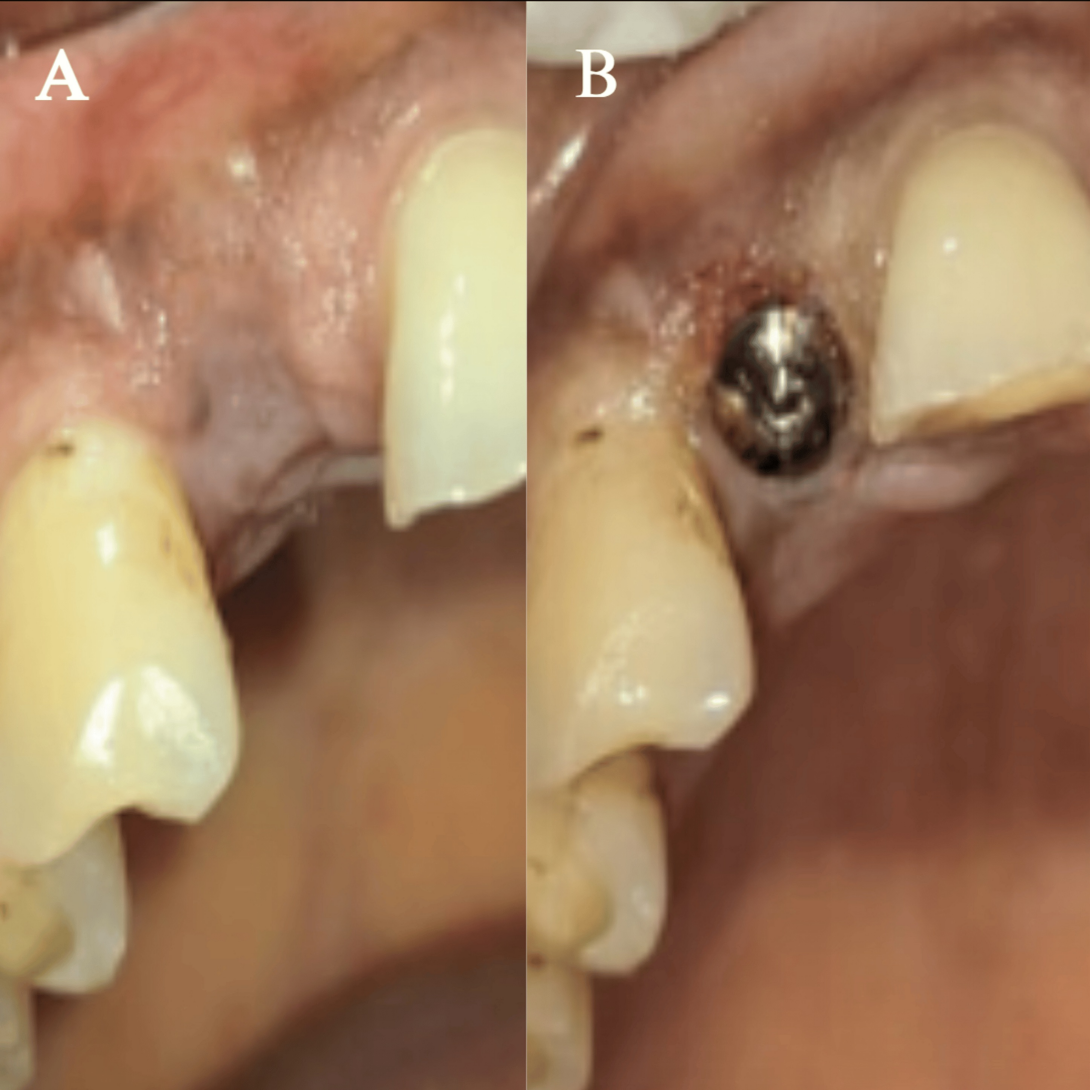
(A) Healing after 3 months; (B) healing cap placement after 3 months (A) shows healing after 3 months post-operative; (B) shows the placement of the healing cap after 3-month recall

After one week with satisfactory healing, suture removal was done (Figure 6). After three months, a CBCT post-operative was done, and a healing cap measuring 4.0 mm was placed. The patient was requested to return one week later for the restorative phase (Figure 7).

Phase 2: Restorative Phase

**Figure 8:**
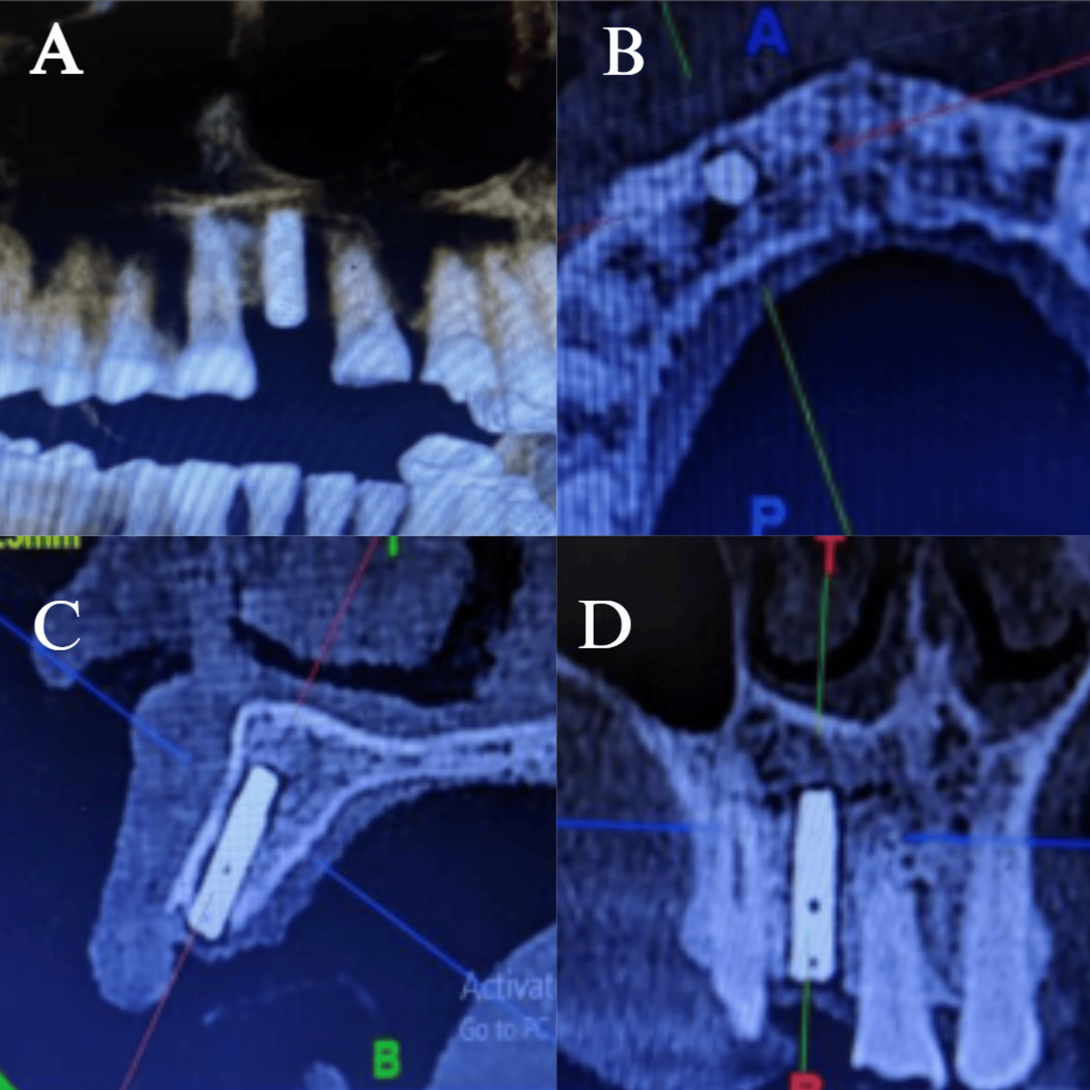
CBCT scans post-implant placement after 3 months (A) shows a panoramic or 3D reconstruction view of the maxilla and mandible. Missing anterior teeth in the upper jaw are visible, demonstrating the edentulous site prepared for implant placement; (B) represents the axial view of the maxilla, where the implant is seen in cross-section. The circular outline corresponds to the implant body within the alveolar bone; (C) is a sagittal section, depicting the implant in its full longitudinal extent within the alveolar ridge; (D) shows a coronal section through the implant site CBCT: cone-beam computed tomography

After clinically effective osseointegration was confirmed using CBCT (Figure 8), a definitive working cast was prepared by taking an open-tray, implant-level definitive impression.

**Figure 9:**
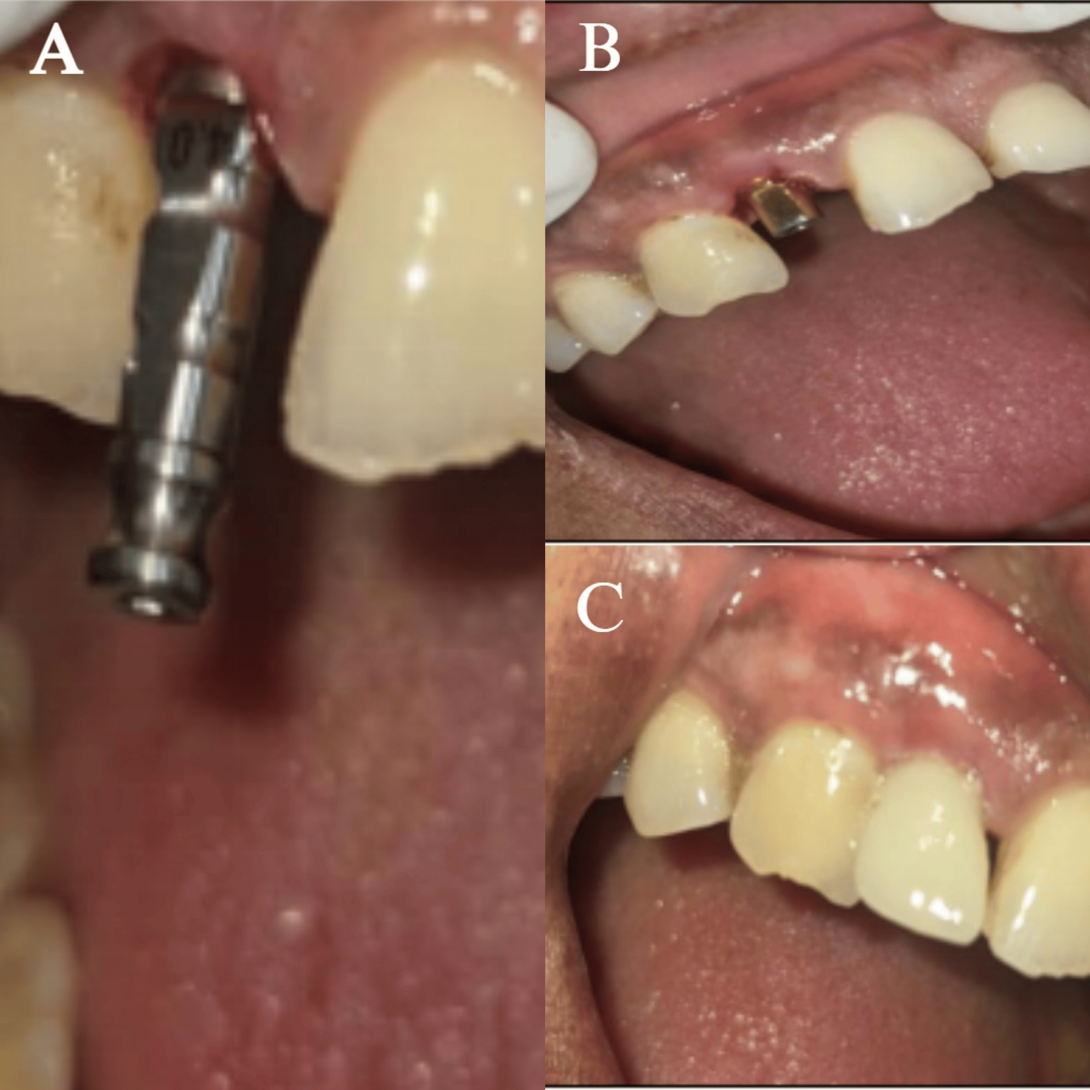
(A) Implant analog irt 12; (B) titanium abutment (UF(II)Narrow Cemented abutment, Ø4.0 mm, C2 mm, H5 mm, Dio Implant, South Korea); (C) dual-core resin was used to bond the zirconia crown to the abutment

After choosing a titanium abutment (UF(II)Narrow Cemented abutment, Ø4.0 mm, C2 mm, H5 mm, Dio Implant, South Korea), CAD-CAM lab technology was used to create an occlusally vented zirconia core that exactly fits the abutment. Once the laboratory work was completed and examined on the dental cast, the abutment-crown assembly was then tried on and screwed into the implant using a 35 Ncm insertion torque. A dual-core resin was used to bond the zirconia crown to the abutment. Occlusal correction was completed and polished (Figure 9).

Phase 3: Follow-Up and Maintenance

The first follow-up was performed after one week, then two weeks, three months, and six months. Patient was educated on maintaining excellent oral hygiene with soft toothbrushes, interdental brushes, and antimicrobial rinses, and was advised to undergo professional cleaning every six months.

## Discussion

The evolution of implant placement techniques has significantly shifted toward methods that prioritize preservation of the alveolar ridge, soft tissue contours, and esthetic outcomes. PET, including the socket-shield technique (SST), has emerged as a viable alternative to conventional immediate implant placement, particularly in the esthetic zone.

Conventional immediate implant placement, while effective, is often accompanied by significant hard and soft tissue resorption, especially of the facial cortical plate, leading to compromised esthetics and implant success. Few studies have highlighted the critical role of the PDL and bundle bone in maintaining socket integrity [2-4]. Since bundle bone is dependent on the presence of a vital PDL, its loss following total extraction leads to inevitable ridge remodeling. The PET approach, particularly SST, involves retaining the buccal/labial aspect of the root to preserve the PDL and associated bundle bone, thereby minimizing post-extraction ridge collapse [7,8]. Studies have shown that this technique helps maintain ridge contour and provides a biologic shield, protecting the facial bone from resorption.

Wu et al. and Ahamed et al. have underscored PET as a biologically driven, minimally invasive approach that maintains ridge volume and optimizes esthetic results [1,6]. Similarly, Bäumer et al. demonstrated, through clinical and volumetric analysis, that SST provides superior preservation of hard tissue over a five-year follow-up period compared to conventional methods [14].

Chen and Buser emphasized that esthetic outcomes in immediate implant placement are largely dependent on the preservation of soft tissue architecture [5]. PET, by maintaining the buccal root segment, supports the soft tissue and avoids collapse of the mid-facial mucosa, enhancing pink esthetics. Multiple comparative studies have reinforced the superiority of SST. Atef et al. give a comparative evaluation of peri-implant soft and hard tissue dimensional changes and conclude that the SST can preserve hard and soft peri-implant tissues following immediate implant placement [12]. Likewise, Abd-Elrahman et al. concluded that SST resulted in less buccal bone resorption and improved esthetic outcomes [13].

From a histological standpoint, PET maintains biologic width and promotes the formation of new cementum and connective tissue adaptation over the retained root segment [8]. Casey and Lauciello and Salama et al. previously introduced the concept of root submergence, which laid the groundwork for PET techniques. Importantly, they also provided additional clinical evidence that socket-shield integration is stable over time and facilitates successful osseointegration of implants placed immediately behind the retained shield [9,10].

Two reviews by Blaschke and Schwass and Salem et al. also provided the evidence that SST showed a more favorable impact on esthetic rehabilitation of anterior teeth compared to conventional immediate implant placement, but this conclusion is based on only a limited number of well-designed prospective randomized controlled trials (RCTs) and non-RCTs [15,16]. Additional RCTs with larger sample sizes, longer follow-up periods, and robust methodology are needed to confirm these findings [15,16].

While the data are promising, the technique requires meticulous execution. Risks include shield exposure, mobility, or infection if not adequately planned and performed. Furthermore, Buser et al. emphasize the importance of anatomical considerations and case selection to achieve predictable results in the anterior maxilla, as in this case [11].

## Conclusions

Although immediate implant placement with the SST as part of PET is becoming increasingly recognized in clinical practice, detailed case reports remain valuable for documenting procedural details, patient selection, and short-term outcomes. This report highlights that PET can achieve stable functional and esthetic results by preserving alveolar ridge dimensions and soft tissue contours, particularly in the anterior maxilla, where soft tissue stability is critical. By retaining part of the root structure, the technique supports the buccal plate and gingival profile, reducing post-extraction remodeling and enhancing implant esthetics. Although encouraging, PET remains an evolving concept, with evidence largely limited to case reports and small clinical series, underscoring the need for prospective trials and randomized controlled studies to establish long-term predictability. Future research should focus on standardized surgical workflows, clear selection criteria, integration of digital planning and guided surgery, and evaluation of patient-reported outcomes including comfort, satisfaction, quality of life, and cost-effectiveness to determine the broader clinical applicability and value of PET.
